# Assessment of Liver and Kidney Safety of Silver Fir (*Abies alba*) Branch Extract: An Open‐Label Human Study of a Dietary Supplement

**DOI:** 10.1002/fsn3.71991

**Published:** 2026-07-14

**Authors:** Katja Schoss, Samo Kreft

**Affiliations:** ^1^ Faculty of Pharmacy, University of Ljubljana Ljubljana Slovenia

**Keywords:** dietary supplements, human safety study, liver and kidney function, polyphenols, safety assessment, silver fir ( *Abies alba* )

## Abstract

Extracts of silver fir ( *Abies alba* ) branches are rich in polyphenolic compounds and are increasingly used as dietary supplements. Despite their growing consumption, systematic human data on their safety remain limited. This study evaluated the effects of a standardized silver fir branch extract on biochemical markers of liver and kidney function in healthy adults. An open‐label, single‐arm human study was conducted in 15 healthy volunteers (eight men and seven women; mean age 48 years). Following a two‐week comparison period, participants consumed 900 mg/day of a standardized silver fir branch extract for 14 days. Serum markers of liver function (AST, ALT, GGT, ALP, and total bilirubin) and kidney function (urea, creatinine, and uric acid) were measured at baseline, after the comparison period, and after supplementation. The study was retrospectively registered at ClinicalTrials.gov. All participants completed the study without reported adverse events. Mean values of all measured liver and kidney markers remained within established reference ranges throughout the study. No statistically significant changes were observed following supplementation, and minor fluctuations were consistent with normal physiological variability. Short‐term consumption of a standardized silver fir branch extract at a daily dose of 900 mg did not adversely affect biochemical markers of liver or kidney function in healthy adults. These findings provide human evidence supporting the short‐term safety of this polyphenol‐rich dietary supplement.

**Trail Registration:** ClinicalTrials.gov: NCT07189143

## Graphical abstract

This study evaluated the short‐term effects of a standardized silver fir ( *Abies alba* ) branch extract (SFBE, Belinal) on biochemical markers of liver and kidney function in healthy adults. No statistically significant or clinically meaningful changes in hepatic or renal biomarkers were observed after 14 days of supplementation with 900 mg/day SFBE.

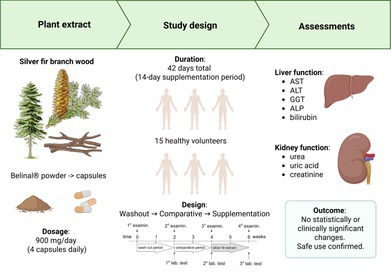

**Abbreviations**
ALP: alkaline phosphatase
ALT: alanine aminotransferase
AST: aspartate aminotransferase
DILI: drug‐induced liver injury
GGT: gamma‐glutamyl transferase
SFBE: silver fir branch extract

## 1 Background

Polyphenols are a diverse group of plant secondary metabolites with well‐documented biological activity. Their health‐promoting properties include antioxidant (Yan et al. 2020), anti‐inflammatory (Zhang and Tsao 2016), cardioprotective (Behl et al. 2020), antidiabetic (de Paulo Farias et al. 2021), neuroprotective (Szwajgier et al. 2017), and chemopreventive (Stagos et al. 2012) effects. These compounds are naturally present in various foods and plants and are widely used as ingredients in dietary supplements due to their potential health benefits (Pandey and Rizvi 2009).

A growing number of dietary supplements now contain high concentrations of polyphenolic compounds. Among these, extracts of silver fir ( *Abies alba* Mill.) (SFBE) have gained attention due to their rich polyphenolic profile and promising pharmacological activity. One such standardized extract is Belinal, which is obtained specifically from the branches of silver fir. The extract contains a complex mixture of polyphenols, mainly lignans, but also flavonoids (e.g., catechin and epicatechin) and phenolic acids (e.g., protocatechinic acid, gallic acid and *p*‐coumaric acid). The lignan fraction contains secoisolariciresinol, isolariciresinol, hydroxymatairesinol, lariciresinol, matairesinol and pinoresinol, compounds known for their antioxidant and enzyme‐modulating effects (Tavčar Benković et al. 2017).

**FIGURE 1:**
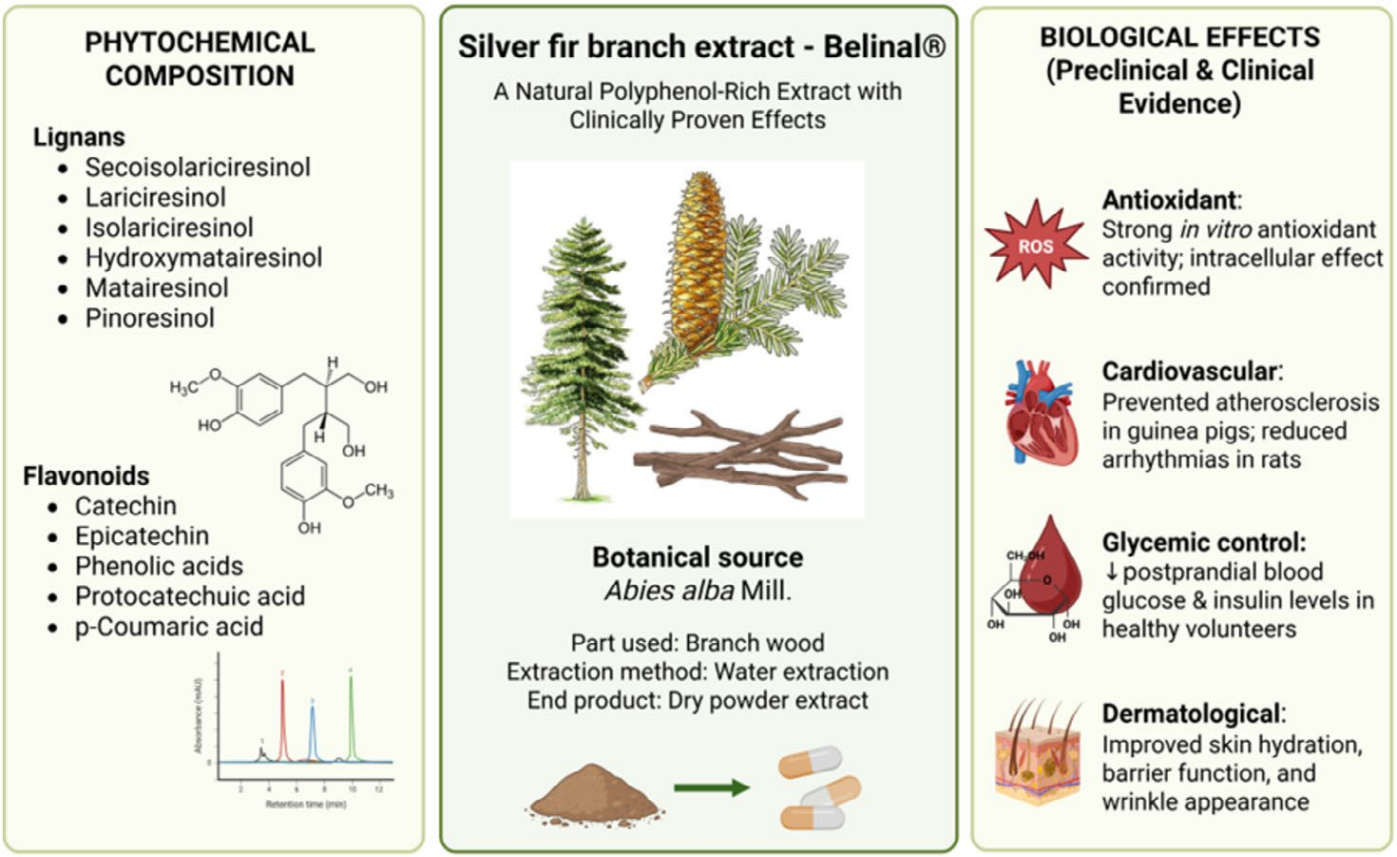
Summary of the phytochemical composition and reported physiological effects of silver fir ( *Abies alba* ) branch wood extract based on previous clinical and laboratory studies.

Several studies have investigated the biological activity of SFBE. In animal models, the extract has shown cardioprotective effects, including vascular protection in atherogenic diet–induced arterial damage and the improved functional response of the ischaemic reperfused heart (Drevenšek et al. 2015, 2016). In vitro studies have also demonstrated inhibition of digestive enzymes involved in glucose metabolism, suggesting potential antidiabetic effects (Lunder et al. 2019). In addition, clinical studies have reported positive effects on postprandial glycaemic control in humans (Debeljak et al. 2016). Despite these promising findings, data on the toxicological safety of SFBE in humans remain limited. The composition and physiological effects of SFBE, as found in previous studies, are summarized in Figure 1.

In the European Union, Belinal was authorized as a traditional food due to the historical use of comparable extracts before 1997, which exempts it from mandatory toxicological testing under the novel food regulations. However, traditional use does not necessarily guarantee safety. Several herbal products with a long history of consumption have later been associated with adverse effects, particularly involving the liver and kidneys, the organs primarily responsible for metabolism and elimination of xenobiotics (Bunchorntavakul and Reddy 2013; Licata et al. 2013).

A well‐known example is green tea ( *Camellia sinensis* ), widely consumed as a beverage and as a concentrated ingredient in dietary supplements. Although green tea has been associated with beneficial metabolic and hepatoprotective effects in experimental studies (Bunchorntavakul and Reddy 2013), several case reports have described hepatotoxic reactions linked to concentrated green tea extracts. These effects have been attributed mainly to high doses of catechins, particularly epigallocatechin gallate, which under certain conditions may induce oxidative stress and mitochondrial dysfunction. Another example is comfrey ( *Symphytum officinale* ), a traditional medicinal plant later found to contain hepatotoxic pyrrolizidine alkaloids. Chronic exposure to these compounds has been associated with progressive liver damage and veno‐occlusive disease (Medicines Agency, European 2016). These cases illustrate that even long‐used herbal products may pose toxicological risks, particularly when consumed in concentrated forms.

For products such as SFBE, which are taken orally and metabolized systemically, the liver and kidneys are the primary target organs for potential toxicity. Hepatotoxicity may be manifested by elevated serum levels of enzymes such as aspartate aminotransferase (AST), alanine aminotransferase (ALT), alkaline phosphatase (ALP), and gamma‐glutamyl transferase (GGT) or elevated bilirubin levels. Nephrotoxicity can be detected by changes in serum levels of urea, creatinine, and uric acid. These biomarkers are widely used in clinical practice as indicators of potential organ toxicity (Kwo et al. 2017).

Although pharmacodynamic studies have investigated the physiological effects of SFBE, these studies were not designed to assess toxicological safety (Debeljak et al. 2016). To date, no clinical study has systematically evaluated the potential effects of SFBE on liver and kidney function in humans. The aim of this study was therefore to evaluate the safety of oral administration of SFBE in healthy adult volunteers by monitoring biochemical markers of liver and kidney function.

## 2 Material and Methods

### 2.1 Study Design and Ethical Approval

The open‐label, single‐arm, prospective clinical study was designed to evaluate the potential effects of a standardized water extract of silver fir ( *Abies alba* ) branch (SFBE) (marketed under the trade name Belinal) on liver and kidney function in healthy adult volunteers.

The study was approved by the National Medical Ethics Committee of the Republic of Slovenia (approval no. 0120–642/2017/5, granted on 12 July 2018). Participant recruitment started on 20 August 2018, and the final study visit took place on 29 October 2018. The approved clinical protocol remained unchanged throughout the study. All procedures were conducted in accordance with the Declaration of Helsinki and applicable national regulations. Written informed consent was obtained from all participants prior to recruitment. Participants were informed about the purpose, design, and methods of the study in a clear and understandable manner. They were also informed that participation was voluntary and that they could withdraw their consent at any time without giving reasons. The study was registered retrospectively at ClinicalTrials.gov (registered on September 23, 2025).

### 2.2 Study Participants

Fifteen healthy adult volunteers (eight men and seven women) aged between 37 and 55 years (mean age: 48) were recruited for the study. The participants received no financial compensation but were provided with the test product and the results of their laboratory tests. Exclusion criteria included: the presence of a known medical condition; the use of medication other than occasional painkillers (participants did not take painkillers during the study); the use of dietary supplements; known mental disorders (e.g., depression and psychosis), severe alcoholism or drug abuse; any condition that would interfere with the participant's ability to comply with the study protocol; consumption of more than two units of alcohol per day (equivalent to 20 g ethanol or approximately 2 dL wine, 5 dL beer or 0.3 dL spirits); and participation in other research studies.

### 2.3 Intervention and Study Procedure

The study lasted a total of 6 weeks and was divided into three consecutive 2‐week phases:

1. *A washout period* (Days 0–14) during which participants were instructed not to use any dietary supplements in order to minimize potential carry over effects from supplement use prior to enrolment,
2. *A comparison period* (Days 14–28), in which the natural variability of the biochemical parameters was assessed, and
3. *A test period* (Days 28–42) during which participants received the study intervention.

During the test period, participants consumed 900 mg of SFBE daily in the form of four capsules of 225 mg each, taken four times daily at evenly spaced times during waking hours. The selected daily dose of 900 mg exceeded the standard recommended dose of 300 mg/day and the commonly used maximum dose of 660 mg/day (“Belinal Product Information, Abies Labs d.o.o.” 2026) for short‐term supplementation of SFBE. This higher dose was intentionally chosen to provide a conservative safety assessment while remaining within ethically acceptable exposure limits. The study product SFBE (Belinal) was manufactured by AbiesLabs d.o.o., Slovenia. Each capsule contained 225 mg of standardized silver fir ( *Abies alba* ) branch extract with ≥ 8% of lignans, ≥ 30% of phenols and standard excipient (maltodextrin), as declared by the manufacturer. The investigated extract originated from production batch 2017Oct‐00325 and contained 68% total phenols and 14% lignans, as specified by the manufacturer. The batch complied with internal quality specifications for heavy metals and microbiological contamination and met the manufacturer's internal specifications and applicable food‐supplement quality requirements. In contrast to some other European countries, the use of plant protection products in forests is not permitted in Slovenia as stipulated in Article 31 of the Slovenian Forest Act; therefore, pesticide residues in the investigated silver fir extract were not analyzed. The extract is obtained by aqueous extraction of silver fir branches at a plant material‐to‐water ratio of approximately 1:5, using extraction temperatures of 70°C–90°C for 30–60 min. The phytochemical profile of the extract was characterized using HPLC with UV detection based on comparison of retention times with available reference standards and literature data, as previously described (Schoss et al. 2026). A representative chromatographic fingerprint illustrating the main lignan constituents of the extract (isolariciresinol, hydroxymatairesinol, secoisolariciresinol, lariciresinol, pinoresinol and matairesinol) is provided in the Supporting Information (Figure S1).

**FIGURE 2:**
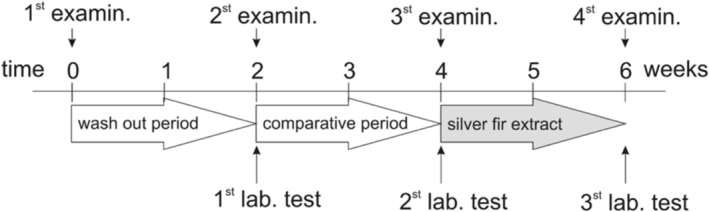
Timeline of the study.

Participants were instructed to maintain their usual dietary and lifestyle habits and not to take any other dietary supplements or make any significant changes to their lifestyle. Compliance assessment was based on participant self‐reporting during scheduled clinical visits, as no formal capsule count verification was performed. Clinical visits took place at the beginning and end of each study phase. Eligibility criteria and informed consent were assessed at the initial visit. Fasting blood samples were collected at baseline (Day 14), at the end of the comparison phase (Day 28), and after the intervention period (Day 42). At the beginning of the intervention phase, participants were instructed to begin supplementation with SFBE (900 mg/day). Adverse event monitoring was based on open questioning during scheduled clinical visits. The entire study procedure is shown in Figure 2.

### 2.4 Laboratory Assessments

The blood samples were collected at three points in time: Day 14 (end of the washout period), Day 28 (end of the comparison period), and Day 42 (end of the test period). All blood samples were collected under fasting conditions and analyzed by Adria Lab d.o.o. (Diagnostični laboratorij, Šestova 2, Ljubljana, Slovenia), part of the SYNLAB Group, a laboratory accredited to ISO 15189, which ensures compliance with international standards for quality management and technical competence. The following biochemical serum parameters were determined: liver function markers: aspartate aminotransferase (AST), alanine aminotransferase (ALT), alkaline phosphatase (ALP), γ‐glutamyl transferase (GGT), total bilirubin and renal function markers: urea, creatinine and uric acid. These biomarkers were chosen because hepatotoxicity and nephrotoxicity are the most relevant potential safety concerns for orally administered botanical extracts, and liver and kidney markers are widely used as sensitive indicators of early or subclinical organ toxicity. Sex‐specific reference intervals from the laboratory were applied and are reported in the footnote to Table 1. Participants were monitored at all clinical visits and instructed to report any adverse symptoms spontaneously or at scheduled visits. Additionally, participants were systematically asked at each visit about the occurrence of any new symptoms or unusual health changes since the previous visit. Vital signs, such as blood pressure and heart rate, were not systematically recorded, as the study focused primarily on biochemical safety markers and all participants were healthy adults without known cardiovascular conditions. Enzyme activities were expressed in μkat/L, in accordance with SI units. For reader convenience, values can be converted to U/L by multiplying by 60 (1 μkat/L = 60 U/L).

**TABLE 1:** Mean values and standard deviations of liver and kidney biochemical parameters measured at baseline (Day 14)—1st tests, after the comparative period (Day 28)—2nd tests, and after SFBE supplementation (Day 42)—3rd tests. Reference ranges are provided for comparison below the table.^a^ No statistically significant changes were observed following intervention.

			AST (μkat/L)	ALT (μkat/L)	GGT (μkat/L)	ALP (μkat/L)	Bilirub. (μmol/L)	Urea (mmol/L)	Creat. (μmol/L)	Uric acid (μmol/L)
Meas.	Average ± SD	1st tests	0.448 ± 0.147	0.460 ± 0.178	0.407 ± 0.217	1.057 ± 0.374	15.143 ± 9.214	4.913 ± 1.158	85.80 ± 14.62	344.5 ± 59.1
		2nd tests	0.417 ± 0.129	0.437 ± 0.212	0.413 ± 0.222	1.130 ± 0.359	13.000 ± 6.433	5.627 ± 1.284	84.80 ± 13.19	333.6 ± 73.7
		3rd tests	0.436 ± 0.124	0.486 ± 0.255	0.431 ± 0.249	1.139 ± 0.412	13.967 ± 9.819	5.793 ± 1.326	85.80 ± 13.17	327.1 ± 74.1
Absolute difference	Difference of average	1st to 2nd	−0.031	−0.023	0.005	0.073	−2.143	0.713	−1.000	−10.9
		2nd to 3rd	0.019	0.049	0.019	0.009	0.867	0.167	1.000	−6.5
	Average of difference	1st to 2nd	−0.034	−0.023	0.005	0.073	−1.7	0.71	−1.0	−10.9
		2nd to 3rd	0.019	0.049	0.019	0.009	0.9	0.17	1.0	−6.5
% change	Difference of average	1st to 2nd	−6.96	−4.93	1.31	6.87	−14.15	14.52	−1.17	−3.15
		2nd to 3rd	4.64	11.13	4.52	0.83	6.67	2.96	1.18	−1.94
	Average of difference	1st to 2nd	−5.38	−3.67	1.46	8.27	−5.05	16.70	−0.82	−3.44
		2nd to 3rd	7.00	10.62	2.74	−0.31	5.10	4.04	1.34	−1.74
*t*‐test	Paired	1st to 2nd	0.163	0.553	0.751	0.012	0.188	0.044	0.466	0.242
	Non‐paired		0.551	0.753	0.947	0.591	0.483	0.121	0.846	0.659
	Paired	2nd to 3rd	0.469	0.141	0.303	0.722	0.551	0.471	0.474	0.474
	Non‐paired		0.679	0.574	0.830	0.948	0.780	0.729	0.837	0.812

*Note:* Women: AST < 0.52 μkat/L; ALT < 0.56 μkat/L; GGT < 0.63 μkat/L; ALP < 1.74 μkat/L; total bilirubin < 17 μmol/L; urea 2.8–8.1 mmol/L; creatinine 44–80 μmol/L; uric acid 143–339 μmol/L. Men: AST < 0.58 μkat/L; ALT < 0.74 μkat/L; GGT < 0.92 μkat/L; ALP < 2.15 μkat/L; total bilirubin < 17 μmol/L; urea 2.8–8.1 mmol/L; creatinine 62–106 μmol/L; uric acid 202–416 μmol/L.

Abbreviations: ALP, alkaline phosphatase; ALT, alanine aminotransferase; AST, aspartate aminotransferase; Bilirub., total bilirubin; Creat., creatinine; GGT, γ‐glutamyl transferase.

^a^ Sex‐specific reference intervals provided by the certified laboratory (Adria Lab d.o.o., SYNLAB Group, ISO 15189 accredited) were applied.

### 2.5 Statistical Analysis

Statistical analyses were conducted using Microsoft Excel 2010 and SPSS. Normal distribution of the data was first confirmed using the Shapiro–Wilk test. Differences in biochemical parameters between study phases were assessed using paired and unpaired Student's *t*‐tests. A *p*‐value < 0.05 was considered statistically significant. Paired analyses were considered primary due to the within‐subject longitudinal design.

The sample size calculation indicated that a minimum of 10 participants was sufficient to achieve a statistical power of 90% (1 − *β* = 0.9), assuming a natural variability of 60% (standard deviation) and a clinically relevant change of 160% (effect size), representing a substantial deviation from normal biological variability. The calculation was performed using an online tool (Schoenfeld 2020). This 160% threshold was used only for sample size estimation; in the actual data analysis, any statistically significant change was considered a potential safety signal. As the primary objective of the study was exploratory safety assessment across a limited set of predefined biomarkers, no formal correction for multiple comparisons was applied. Potential safety signals were evaluated based on both statistical significance and clinical relevance, including consistent worsening across multiple hepatic or renal biomarkers or marked deviations from established reference intervals.

## 3 Results

### 3.1 Baseline Characteristics

A total of 15 healthy adult volunteers (eight men and seven women, aged 37–55 years) were enrolled and completed the study, with a mean age of 48 ± 3.8 years. All participants were free from known medical conditions; baseline laboratory parameters were generally within or near reference ranges, although isolated values outside the reference intervals were observed in some participants (Detailed individual values are provided in Data S1).

All participants adhered to the prescribed dosing regimen of SFBE (Belinal) (900 mg/day) during the 14‐day test period, and no dropouts occurred. The intake of other supplements or medications was restricted, and no analgesics were used during the study.

At baseline (Day 14), after the washout period, all measured liver and kidney function parameters (AST, ALT, GGT, ALP, total bilirubin, urea, creatinine, and uric acid) were generally within the respective reference ranges (Table 1). In a small number of participants, isolated values exceeded the reference intervals (e.g., bilirubin or creatinine). These findings were reviewed during the screening assessment in the context of the participant's medical history and clinical evaluation. Overall, at least one parameter was outside the reference range in nine of the 15 participants at baseline.

### 3.2 Variability of Biochemical Parameters During the Comparison Period

The comparison period (Days 14 to 28), during which no food supplement was administered, was used to assess the natural physiological variability of the laboratory parameters. A paired *t*‐test, accounting for repeated measurements within the same individuals, showed a statistically significant decrease in alkaline phosphatase (ALP) (*p* = 0.012) and an increase in urea (*p* = 0.044) (Table 1). These minor changes remained within the reference ranges.

In contrast, a non‐paired *t*‐test, which does not account for individual adjustment, showed no significant differences. All observed changes, although statistically significant, were not considered clinically meaningful and remained within reference values.

Consistent with this variability, the number of participants with at least one parameter outside the reference range decreased from nine at baseline to eight on Day 28.

### 3.3 Effects of SFBE Supplementation on Liver and Kidney Parameters

No adverse events were reported during the study.

Of the 360 planned measurements (eight parameters × 15 participants × three time points), three could not be performed due to insufficient sample quality (two in the first analysis set and one in the second). These missing values did not affect the integrity of the dataset or the overall conclusions of the study.

After administration of SFBE from Days 28 to 42, no statistically significant changes were observed in any of the biochemical markers tested (AST, ALT, GGT, ALP, total bilirubin, urea, creatinine, and uric acid; paired *t*‐test, all *p* > 0.05) (Table 1). Mean values remained within the reference ranges, and no clinically meaningful changes were observed during the intervention period. Mean changes between Days 28 and 42 together with 95% confidence intervals are presented in Supporting Information Table S2.

At the third test (Day 42), nine of the 15 participants had at least one parameter outside the reference range, consistent with the numbers observed at baseline (nine at Day 14 and eight at Day 28). The total number of out‐of‐range measurements was 15, 16, and 15 at the first, second, and third time points, respectively. One participant showed an isolated increase in uric acid (399 and 387 μmol/L in the first two tests; 441 μmol/L in the third test), which was not statistically significant and likely reflects normal physiological variation.

A summary of all measured parameters and their mean values across the study time points is provided in Table 1, while detailed individual data are presented in Supporting Information (Table S1).

## 4 Discussion

### 4.1 Effects of SFBE on Liver and Kidney Function

The results of this study indicate that SFBE was well tolerated in the population studied. As expected in a healthy cohort, interindividual variability in biochemical parameters was observed. Mildly elevated bilirubin values in otherwise asymptomatic individuals without accompanying liver enzyme abnormalities may reflect benign conditions such as Gilbert syndrome or normal physiological variability. Similarly, isolated creatinine values near the upper reference limit may be influenced by factors such as hydration status, physical activity, or muscle mass. None of the observed baseline patterns suggested clinically relevant hepatic or renal disease. Sensitivity analyses excluding participants with baseline bilirubin, ALT, or creatinine values above the reference intervals yielded unchanged findings. These isolated deviations are consistent with normal biological variability, including influences such as diet, fluid intake, and circadian rhythms.

The comparison phase provided important context for interpreting the intervention results. The observed intraindividual fluctuations during this period highlight the importance of paired analyses in longitudinal studies, as within‐subject comparisons offer greater sensitivity for detecting subtle changes. In this respect, the comparison phase served as an internal control, allowing differentiation between natural physiological variability and potential intervention‐related effects, which is particularly relevant in the absence of a placebo group.

During the intervention period, no statistically significant or clinically meaningful changes were observed in any assessed biochemical markers. Mean values remained within reference ranges, and no patterns indicative of hepatotoxicity or nephrotoxicity were detected.

The stability of liver enzymes, particularly ALT and AST as sensitive markers of hepatocellular stress, represents an important finding. Combined assessment of aminotransferases (ALT and AST) and cholestatic markers (ALP and GGT) enables differentiation between hepatocellular and cholestatic patterns of liver injury. While ALT and AST primarily reflect hepatocellular damage, elevations in ALP and GGT are more typically associated with cholestatic or biliary dysfunction. In addition, the relationship between ALT and ALP can be evaluated using the R ratio, which allows classification of liver injury as hepatocellular, cholestatic, or mixed (Kwo et al. 2017). In the present study, all these markers remained stable, indicating no biochemical pattern suggestive of liver injury. No consistent pattern of worsening across hepatic or renal biomarkers was observed during the intervention period. No biochemical pattern suggestive of hepatocellular, cholestatic, or renal toxicity was identified. Renal function markers (creatinine and urea) also remained unchanged, further supporting the absence of nephrotoxic effects.

The absence of detectable changes in liver and kidney function markers suggests that SFBE did not cause hepatotoxic or nephrotoxic effects under the conditions tested. This is particularly relevant for herbal supplements, as cases of liver and kidney injury have been reported, especially with concentrated extracts (Bunchorntavakul and Reddy 2013; Licata et al. 2013; Bonkovsky 2006).

The present findings are consistent with previous studies investigating SFBE. Although these studies primarily focused on pharmacodynamic or physiological outcomes, they also reported good tolerability and did not indicate relevant safety concerns. For example, a randomized, double‐blind, cross‐over human study demonstrated that supplementation with SFBE (Belinal) reduced the postprandial glycaemic response in healthy volunteers, lowering the area under the glucose curve by approximately 35% compared with placebo, without reported adverse events (Debeljak et al. 2016). Similarly, preclinical studies have demonstrated beneficial cardiovascular effects, including improved vascular relaxation and reduced atherosclerotic plaque formation (Drevenšek et al. 2015), as well as cardioprotective effects in ischemia–reperfusion models, with reductions in arrhythmia duration of up to approximately 80% (Drevenšek et al. 2016). Together, these findings suggest that the biological activity of silver fir extracts is associated primarily with antioxidant and vascular protective mechanisms rather than toxicological effects.

Although these studies were not designed to assess toxicological endpoints, they provide additional contextual support for the present findings. The dose used in this study (900 mg/day for 14 days) exceeded both the standard recommended dose (300 mg/day) and commonly used short‐term maximum doses (660 mg/day). This higher dose was selected to provide a conservative safety assessment while remaining within ethically acceptable limits. The absence of adverse effects under these conditions further supports the safety of SFBE for short‐term use.

Belinal is a standardized water extract of silver fir ( *Abies alba* ) branches that has been available on the European market as a food supplement since 2014. Its regulatory status is defined under Directive 2002/46/EC, which classifies food supplements as concentrated sources of substances with nutritional or physiological effects, marketed in measured doses. Based on its composition and dosage form, Belinal is classified as a food supplement (“Directive–2002/46/EC‐EN‐EUR‐Lex” 2025).

Although preclinical toxicological testing is not mandatory for food supplements under this framework, products must comply with general food safety principles. In addition, novel foods require evaluation under Regulation (EU) 2015/2283 (European Commission 2015). In the case of Belinal, historical evidence of prior use of comparable silver fir extracts before 1997 supported its classification as a nonnovel food, and no additional authorisation was required.

Nevertheless, traditional use alone does not guarantee safety, especially when botanicals are consumed as concentrated extracts. Several herbal products, including green tea and comfrey, have been associated with hepatotoxic or nephrotoxic effects under certain conditions (Licata et al. 2013; Stickel and Shouval 2015; Navarro and Lucena 2014). These examples highlight the importance of systematic safety evaluation.

The liver and kidneys play a central role in xenobiotic metabolism and elimination, making them primary targets of toxicity. Even without clinical symptoms, subclinical damage can be detected through laboratory biomarkers. Therefore, assessment of biochemical parameters such as ALT, AST, GGT, ALP, urea, and creatinine represents a standard and relevant approach in the safety evaluation of botanical supplements (Khan et al. 2022; Rivero‐Pino and Casanova 2023).

Although SFBE has previously been investigated in a human study assessing postprandial glucose and insulin responses, that study was not designed to evaluate liver or kidney safety. The present study therefore addresses an important gap by providing structured clinical data on hepatorenal safety.

Overall, the findings of this study contribute to the safety profile of SFBE by demonstrating no detectable hepatotoxic or nephrotoxic effects during short‐term use in healthy individuals, consistent with current expectations for clinical safety evaluation of botanical food supplements.

### 4.2 Study Strengths and Limitations

A key strength of this study is its three‐phase design, which includes a comparative period that enables the assessment of natural biological variability. The use of objective laboratory endpoints increases reliability, and the relatively high dosage enhances the study's sensitivity to detect adverse effects.

The study population consisted of healthy adult volunteers. This group was selected because SFBE is marketed and intended as a food supplement for generally healthy individuals seeking additional support for well‐being. Accordingly, healthy adults represent the primary target population for safety evaluation, rather than patients with existing hepatic or renal disorders.

However, several limitations should be noted, including the small sample size (*n* = 15), the relatively short intervention duration (14 days), which reflects a short‐term safety assessment focusing on early biochemical indicators of hepatotoxicity and nephrotoxicity, and the inclusion of only healthy adults. Another limitation is that the study examined a limited set of commonly used biochemical markers of liver and kidney function (AST, ALT, GGT, ALP, bilirubin, urea, creatinine, and uric acid). Although these parameters are widely accepted as primary indicators of hepatorenal safety, additional biomarkers could provide a more comprehensive evaluation of systemic safety. In particular, electrolyte measurements such as sodium and potassium may offer further insight into renal tubular function and electrolyte homeostasis, but these parameters were not included in this study. Additionally, compliance assessment relied on participant self‐reporting and did not include formal capsule count verification. Future research would benefit from including a broader range of biomarkers, longer intervention periods, and more diverse participant populations to better assess potential long‐term and population‐specific effects.

## 5 Conclusion

This study provides clinical data indicating that oral administration of the standardized water extract of silver fir ( *Abies alba* ) branch (SFBE) did not produce clinically meaningful changes in selected biochemical markers of liver and kidney function in healthy adult volunteers. No statistically significant changes in serum levels of liver enzymes, bilirubin, or indicators of renal function such as urea, creatinine, and uric acid were observed during 14 days of supplementation with a daily dose of 900 mg. Furthermore, no adverse events were reported by participants, and the measured values remained within reference ranges or showed only random fluctuations.

Taken together, these findings indicate that short‐term supplementation with SFBE at a dose of 900 mg/day was well tolerated under the conditions of this study. Given its long‐standing use as a dietary supplement and its phytochemical profile rich in polyphenols, compounds typically associated with hepatoprotective and nephroprotective properties, no biochemical indications of hepatotoxic or nephrotoxic effects were observed in the investigated healthy adult population. However, due to the relatively small sample size and short duration of the intervention, further studies involving larger and more diverse populations and longer exposure periods are needed to confirm the long‐term safety profile of SFBE and to evaluate its use in specific populations, such as individuals with preexisting liver or kidney disease.

## Author Contributions

**Katja Schoss:** writing – original draft, writing – review and editing, visualization. **Samo Kreft:** conceptualization, methodology, validation, formal analysis, resources, writing – original draft.

## Funding

The laboratory analyses were funded by the manufacturer of SFBE (Belinal). The sponsor had no role in study design, data collection, statistical analysis, interpretation of results, or writing of the manuscript.

## Ethics Statement

The study was approved by the National Medical Ethics Committee at the Ministry of Health of the Republic of Slovenia (approval No. 0120–642/2017/5, 12 July 2018). The study was conducted in accordance with the principles of the Declaration of Helsinki.

## Consent

All participants provided written informed consent prior to their inclusion in the study and for publication of data and results.

## Conflicts of Interest

K.S. and S.K. disclose that the costs of laboratory testing were covered by AbiesLabs, the producer of Belinal. The authors declare no conflicts of interest.

## Supporting information


**Figure S1:** Representative HPLC chromatogram of the standardized silver fir ( *Abies alba* ) branch extract used in the study, illustrating the phytochemical fingerprint of the extract. The main lignan constituents are indicated as follows: (1) isolariciresinol, (2) hydroxymatairesinol, (3) secoisolariciresinol, (4) lariciresinol, (5) pinoresinol, and (6) matairesinol.
**Table S1:**. Individual biochemical parameters at all study timepoints.
**Table S2:**. Mean changes between Days 28 and 42 with corresponding 95% confidence intervals.

## Data Availability

Individual participant laboratory values are provided in Supporting Information. The complete dataset generated and analyzed during the current study is available from the corresponding author on reasonable request.
