# Supplementary material for: Assessment of Liver and Kidney Safety of Silver Fir (Abies alba) Branch Extract: An Open‐Label Human Study of a Dietary Supplement
Source: Food Sci Nutr. 2026 Jul 14;14(7):e71991. doi: 10.1002/fsn3.71991 (PMC13367129; doi:10.1002/fsn3.71991)
Supplement: Supplementary file 1 — Figure S1: Representative HPLC chromatogram of the standardized silver fir ( Abies alba ) branch extract used in the study, illustrating the phytochemical fingerprint of the extract. The main lignan constituents are indicated as follows: (1) isolariciresinol, (2) hydroxymatairesinol, (3) secoisolariciresinol, (4) lariciresinol, (5) pinoresinol, and (6) matairesinol. Table S1:. Individual biochemical parameters at all study timepoints. Table S2:. Mean changes between Days 28 and 42 with corresponding 95% confidence intervals. [file FSN3-14-e71991-s001.docx]

**Supplementary Material**

**Table S1. Individual Biochemical Parameters at All Study Timepoints:**

This table contains individual participant values for all measured liver and kidney function parameters (AST, ALT, GGT, ALP, total bilirubin, urea, creatinine, uric acid) at three study timepoints: day 14 (baseline), day 28 (post-comparative period), and day 42 (post-supplementation). Each column represents a unique participant and the corresponding laboratory values at three study visits. This dataset enables detailed tracking of intra-individual changes over time and supports transparency of the reported results. All values were measured by a certified diagnostic laboratory under standardized conditions. n.a. = not available.

|  | No. | 1 | 2 | 3 | 4 | 5 | 6 | 7 | 8 | 9 | 10 | 11 | 12 | 13 | 14 | 15 |
| --- | --- | --- | --- | --- | --- | --- | --- | --- | --- | --- | --- | --- | --- | --- | --- | --- |
|  | Sex | M | M | W | M | W | W | W | W | M | M | M | M | W | M | W |
| **First tests**  **(baseline)** | **AST** | 0.39 | 0.41 | 0.78 | 0.39 | 0.23 | 0.45 | 0.48 | 0.45 | 0.35 | 0.40 | n.a. | 0.47 | 0.41 | 0.32 | 0.74 |
|  | **ALT** | 0.32 | 0.36 | 0.26 | 0.35 | 0.23 | 0.55 | 0.56 | 0.66 | 0.31 | 0.45 | 0.56 | 0.34 | 0.56 | 0.49 | 0.9 |
|  | **GGT** | 0.53 | 0.28 | 0.23 | 0.21 | 0.18 | 0.43 | 0.14 | 0.26 | 0.27 | 0.30 | 0.81 | 0.77 | 0.48 | 0.60 | 0.62 |
|  | **ALP** | 1.2 | 0.88 | 1.04 | 0.52 | 0.72 | 0.91 | 0.73 | 0.97 | 1.02 | 1.31 | 0.95 | 1.94 | 1.74 | 0.85 | 1.08 |
|  | **bilirubin** | 44 | 17 | 8 | 19 | 8.0 | 10.0 | 12 | 12 | 15 | 9 | n.a. | 20 | 13 | 9 | 16 |
|  | **urea** | 3.9 | 4.6 | 3.9 | 8.0 | 4.2 | 4.8 | 3.8 | 4.2 | 6.1 | 5.4 | 6.1 | 4.3 | 4.1 | 5.7 | 4.6 |
|  | **creatinine** | 86 | 82 | 77 | 114 | 75 | 71 | 73 | 76 | 105 | 102 | 69 | 91 | 75 | 107 | 84 |
|  | **uric acid** | 283 | 330 | 356 | 359 | 286 | 416 | 273 | 294 | 399 | 264 | 339 | 457 | 355 | 422 | 334 |
| **Second tests**  **(post-comparative period)** | **AST** | 0.38 | 0.45 | 0.67 | 0.47 | 0.24 | 0.34 | 0.29 | 0.31 | 0.36 | 0.34 | 0.45 | 0.38 | 0.46 | 0.40 | 0.71 |
|  | **ALT** | 0.35 | 0.43 | 0.21 | 0.40 | 0.22 | 0.41 | 0.27 | 0.37 | 0.30 | 0.31 | 0.68 | 0.34 | 0.57 | 0.74 | 0.96 |
|  | **GGT** | 0.50 | 0.27 | 0.24 | 0.20 | 0.18 | 0.48 | 0.15 | 0.25 | 0.27 | 0.30 | 0.80 | 0.64 | 0.50 | 0.60 | 0.81 |
|  | **ALP** | 1.28 | 1.07 | 1.07 | 0.62 | 0.78 | 1.05 | 0.79 | 0.86 | 1.02 | 1.48 | 0.97 | 1.97 | 1.66 | 1.01 | 1.32 |
|  | **bilirubin** | 32 | n.a. | 9 | 11 | 7 | 7 | 15 | 9 | 15 | 13 | 9 | 19 | 11 | 11 | 14 |
|  | **urea** | 5.2 | 4.5 | 4.5 | 5.6 | 4.6 | 4.6 | 4.0 | 6.5 | 6.7 | 6.0 | 7.6 | 4.2 | 6.3 | 8.3 | 5.8 |
|  | **creatinine** | 83 | 78 | 73 | 102 | 80 | 71 | 69 | 81 | 97 | 99 | 73 | 95 | 73 | 112 | 86 |
|  | **uric acid** | 285 | 287 | 313 | 279 | 233 | 392 | 274 | 269 | 387 | 277 | 358 | 468 | 356 | 480 | 346 |
| **Third tests**  **(post-supplementation)** | **AST** | 0.40 | 0.36 | 0.63 | 0.56 | 0.24 | 0.38 | 0.29 | 0.50 | 0.36 | 0.36 | 0.52 | 0.32 | 0.66 | 0.45 | 0.51 |
|  | **ALT** | 0.32 | 0.35 | 0.20 | 0.51 | 0.24 | 0.51 | 0.21 | 0.73 | 0.33 | 0.37 | 0.70 | 0.25 | 0.82 | 0.75 | 1.00 |
|  | **GGT** | 0.58 | 0.26 | 0.22 | 0.20 | 0.16 | 0.46 | 0.16 | 0.24 | 0.28 | 0.3 | 0.71 | 0.77 | 0.65 | 0.56 | 0.92 |
|  | **ALP** | 1.39 | 1.09 | 1.12 | 0.53 | 0.74 | 0.97 | 0.74 | 0.84 | 1.06 | 1.32 | 1.05 | 2.01 | 1.91 | 0.94 | 1.38 |
|  |  |  |  |  |  |  |  |  |  |  |  |  |  |  |  |  |
|  | **bilirubin** | 40 | 13 | 11 | 8 | 10 | 10 | 10 | 8 | 12 | 13 | 8 | 35 | 9 | 13 | 8 |
|  | **urea** | 4.9 | 4.5 | 5.0 | 8.0 | 3.9 | 5.7 | 4.1 | 6.0 | 6.5 | 6.6 | 7.1 | 5.4 | 5.5 | 8.4 | 5.3 |
|  | **creatinine** | 85 | 80 | 71 | 102 | 76 | 70 | 72 | 78 | 104 | 101 | 82 | 105 | 74 | 101 | 86 |
|  | **uric acid** | 288 | 295 | 300 | 249 | 235 | 397 | 257 | 268 | 441 | 265 | 410 | 454 | 353 | 394 | 301 |

Abbreviations: AST, aspartate aminotransferase; ALT, alanine aminotransferase; GGT, γ-glutamyl transferase; ALP, alkaline phosphatase; Bilirub., total bilirubin; Creat., creatinine.

AST, ALT, GGT, and ALP are expressed in µkat/L; total bilirubin, creatinine, and uric acid in µmol/L; urea in mmol/L.


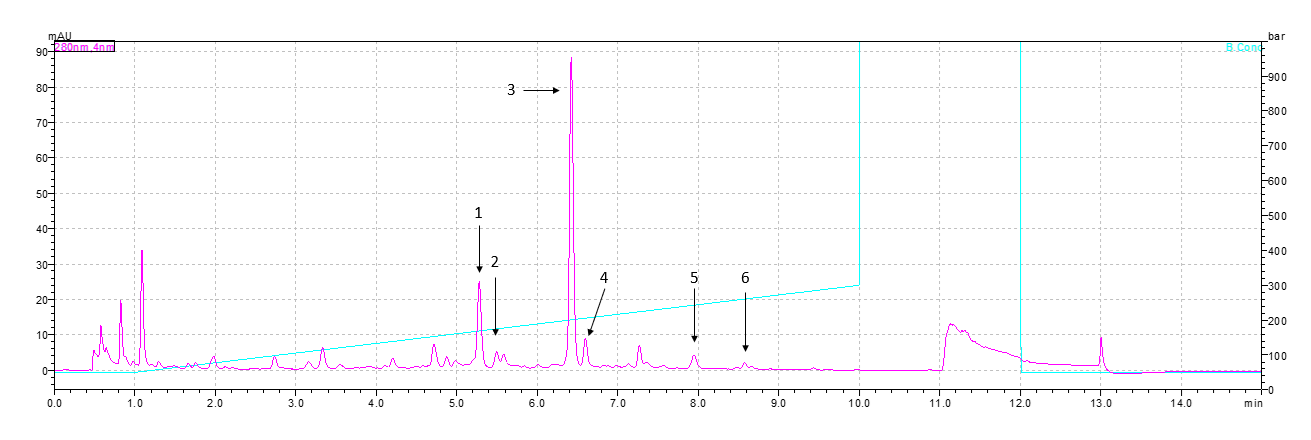


Figure S1. Representative HPLC chromatogram of the standardized silver fir (*Abies alba*) branch extract used in the study, illustrating the phytochemical fingerprint of the extract. The main lignan constituents are indicated as follows: (1) isolariciresinol, (2) hydroxymatairesinol, (3) secoisolariciresinol, (4) lariciresinol, (5) pinoresinol, and (6) matairesinol.

**Table S2. Mean changes between day 28 and day 42 with corresponding 95% confidence intervals.**

| **Parameter** | **Mean change (Day 28 → Day 42)** | **95% CI** |
| --- | --- | --- |
| AST | 0.019 | −0.033 to 0.072 |
| ALT | 0.049 | −0.015 to 0.112 |
| GGT | 0.019 | −0.017 to 0.054 |
| ALP | 0.009 | −0.043 to 0.062 |
| Total bilirubin | 0.929 | −2.045 to 3.902 |
| Urea | 0.167 | −0.290 to 0.623 |
| Creatinine | 1.000 | −1.758 to 3.758 |
| Uric acid | -6.467 | −24.299 to 11.365 |

Mean changes were calculated as day 42 minus day 28 values.

AST, ALT, GGT, and ALP are expressed in µkat/L; total bilirubin, creatinine, and uric acid in µmol/L; urea in mmol/L.
